# Comparison of direct cortical stimulation and transcranial magnetic stimulation in brain tumor surgery: systematic review and meta analyses

**DOI:** 10.1007/s11060-023-04378-4

**Published:** 2023-07-12

**Authors:** Rr. Suzy Indharty, Iskandar Japardi, Rr. Sinta Irina, Steven Tandean, Andre Marolop Pangihutan Siahaan, Michael Lumintang Loe, Alvin Ivander

**Affiliations:** 1 Department of Neurosurgery, Faculty of Medicine, Universitas Sumatera Utara, Dr. Mansyur St. No. 5, Padang Bulan, Medan Baru, Medan, Sumatera Utara 20155 Indonesia; 2 Anesthesiology and Intensive Care Study Program, Universitas Sumatera Utara, Medan, Indonesia; 3 Department of Neurosurgery, Faculty of Medicine, University of Palangka Raya, Palangkaraya, Indonesia; 4 Department of Neurosurgery, Siloam Hospital, Medan, Indonesia; 5 Faculty of Medicine, Universitas Sumatera Utara, Medan, Indonesia

**Keywords:** Transcranial magnetic stimulation, Direct cortical stimulation, Brain tumor, Eloquent area

## Abstract

**Introduction:**

Brain malignancy and, at the same time central nervous system malignancy are two of the most difficult problems in the oncology field of practice. Brain tumors located near or within eloquent areas may represent another challenge toward neurosurgeon treatment. As such, electrical stimulation, either directly or through other methods, may prove necessary as proper mapping of the eloquent area thus may create a proper resection guide. Minimal resection will hopefully preserve patient neurological function and ensure patient quality of life.

**Methods:**

This research is a systematic review and meta-analysis that aim to compare outcomes, primarily adverse event analysis, between direct cortical stimulation and transcortical magnetic stimulation.

**Results:**

Fourteen studies were identified between 2010 and the 2023 interval. While this number is sufficient, most studies were not randomized and were not accompanied by blinding. Meta-analysis was then applied as a hypothesis test, which showed that TMS were not inferior compared to DCS in terms of motoric and lingual outcome which were marked subjectively by diamond location and objectively through a p-value above 0.05.

**Conclusion:**

TMS is a noninvasive imaging method for the evaluation of eloquent brain areas that is not inferior compared to the invasive gold-standard imaging method (DCS). However its role as adjuvant to DCS and alternative only when awake surgery is not available must be emphasized.

## Introduction

Brain malignancy and, at the same time, central nervous system malignancy are two of the most difficult problems in the oncology field of practice. According to the Global Cancer Observatory (GLOBOCAN), tumors affecting the brain and central nervous system were relatively rare, with only 308.102 new cases globally. CNS tumors do cause significant mortality, with 251.329 deaths annually, which means that nearly two-quarters of brain malignancy patients don’t survive [1]. Brain tumors can either emerge primarily as meningiomas or gliomas or secondarily through intracranial metastases of systemic cancer. Such cases may occur anywhere along the central nervous system, and all brain tumor cases would require complex multidisciplinary care involving a neurosurgeon, radiation oncologist, and medical oncologist [2].

Brain tumors present a multitude of challenges that need addressing. In essence, malignant brain tumors by themselves already had a devastating mortality effect, with 2- and 5-year survival rates as low as 36.2 and 27.6%, respectively [3]. Not only that brain tumor directly cause mortality, as it interferes with neurology system, it affects patient quality of life. Symptoms such as fatigue, sleep disorders, and cognitive dysfunction, as well as neurological deficits, were not uncommon [4]. These symptoms may correlate directly with the location of a brain tumor.

Brain tumors located near or within eloquent areas may represent another challenge for neurosurgeons' treatment. It is well known that resection of these lesions may induce permanent post-operative neurological deficits [5]. Eloquent cerebral structures are defined as areas of the brain with readily identifiable neurological function in which injury results in disability. Usually, neuro-oncology damage to the eloquent structure may happen in three scenarios, such as tumor metastases or infiltration into the cortical and subcortical eloquent areas; the effect of resection; and the effect of non-surgical interventions that have a devastating effect on normal cells, such as chemotherapy or radiotherapy [6].

As such, electrical stimulation, either directly or through other methods, may prove necessary as proper mapping of the eloquent area may create a proper resection guide. Minimal resection will hopefully preserve patient neurological function and ensure patient quality of life. Intraoperative electrical stimulation, commonly designated as "direct cortical stimulation (DCS)" is the most sensitive method for eloquent area mapping and has always been deemed the gold standard [7].

On the other hand, transcranial magnetic stimulation (TMS) is also available as a new alternative. While DCS is highly reliable, it is not without its own limitations, especially due to its invasive nature. Due to those fact, neurological mapping in a more non-invasive method such as magnetic resonance imaging or transcranial magnetic stimulation may have a promise with efficacy that is not inferior to DCS as mapping remains a vital part of surgery planning and preparation. Our study will try to compare clinical outcomes between patients who undergo DCS compared with those who undergo TMS mapping [8].

## Methods

### Overview

This research is a systematic review and meta-analysis that aim to compare outcomes, primarily adverse event analysis, between direct cortical stimulation and transcortical magnetic stimulation. Our systematic review adheres to PRISMA guidelines and will seek to answer clinical problems as defined in Table 1.

**Table 1:** Study clinical problem

Patients	Patients with brain tumors
Intervention	Transcortical magnetic stimulation
Comparison	Direct cortical stimulation
Outcome	Safety outcome and adverse effect (e.g.: aphasia, paresis)

### Literature collection

Literature will be obtained either through search engines such as Google Scholar and PubMed or through study references/bibliography review. Study results will be found in search engines through MeSH keywords using a Boolean formula as below: “direct cortical stimulation,” “transcranial magnetic stimulation,” and “brain tumor." The study will undergo title and abstract screening. After title and abstract screening, selected literature will be examined. A full-text examination will attempt to find studies that fulfill certain inclusion and exclusion criteria. The inclusion criteria of the study were limited to a comparative study comparing direct cortical stimulation and transcranial magnetic stimulation in brain tumor patients from the perspective of clinical outcome. A case report, case series, or study where both DCS and TMS were done on the same patient were excluded. To ensure that the evidence level can be considered new and relevant, only studies from 2010 until the 2023 period were included in this study.

### Quality assessment

Study quality assessment will be done through subjective and objective methods. All possible studies will be collected by AI. Title and abstract screenings will also be performed by AI. Then a full-text examination will be performed by RSI, ST, IJ, and AI. If disagreement occurs during the process, IJ will decide the study's eligibility after considering perspectives from all authors. While objective assessment doesn’t necessarily exclude studies, it is still performed to assess study quality and infer possible limitations. Objective quality assessment will be performed with the ROBINS-1 tool as provided by Cochrane.

### Meta-analysis

Meta-analyses will be performed using Review Manager 5.4. Effects that can be assessed statistically will be inputted into the software. The analysis will comprise a homogeneity test. The I^2^ test was used to determine study homogeneity. If the study was homogenous, fixed-effect analysis would be used, and if the study was heterogenous, random effect analysis would be used. All analysis shall be presented in a Forrest plot, and a z-test will be used to determine the hypotheses' ruling. To ensure no publication bias was present in the study, a funnel plot was created and analyzed properly.

## Results

**Fig. 1:**
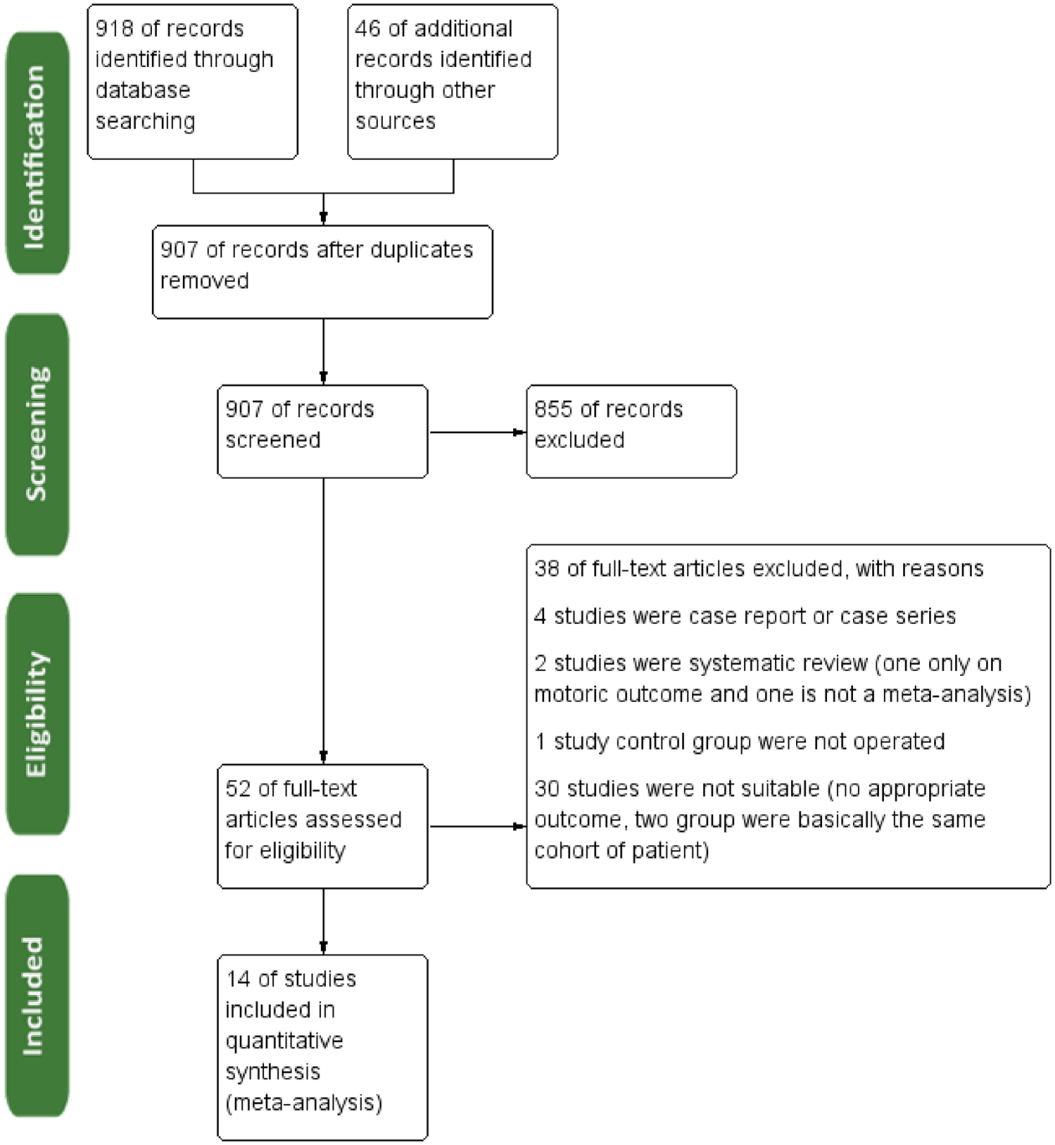
Study flow diagram

Nine hundred and eighteen studies were collected from Google Scholar and PubMed search engines, and upon inspection, 46 studies were collected from bibliographical sources during full-text screening. From those studies, 907 records remained after duplicates were removed. Specifically, 855 studies were not eligible during abstract and title screening and were thus excluded immediately. From 52 articles that underwent an eligibility study with a full text study, 30 studies were not comparative studies comparing clinical outcomes; 1 study had a control group in which operation was not performed in all cohort; 4 studies were either case series or case reports; and 2 study was a systematic review; one is not a meta-analysis while the other only assessed motoric outcome, confirming that this is a one-of-a-kind study performed recently. Afterward, 14 eligible studies will undergo objective assessment with the ROBINS-1 tool and undergo meta-analysis regardless of the objective assessment result. All of this process can be observed in Fig. 1.

**Fig. 2:**
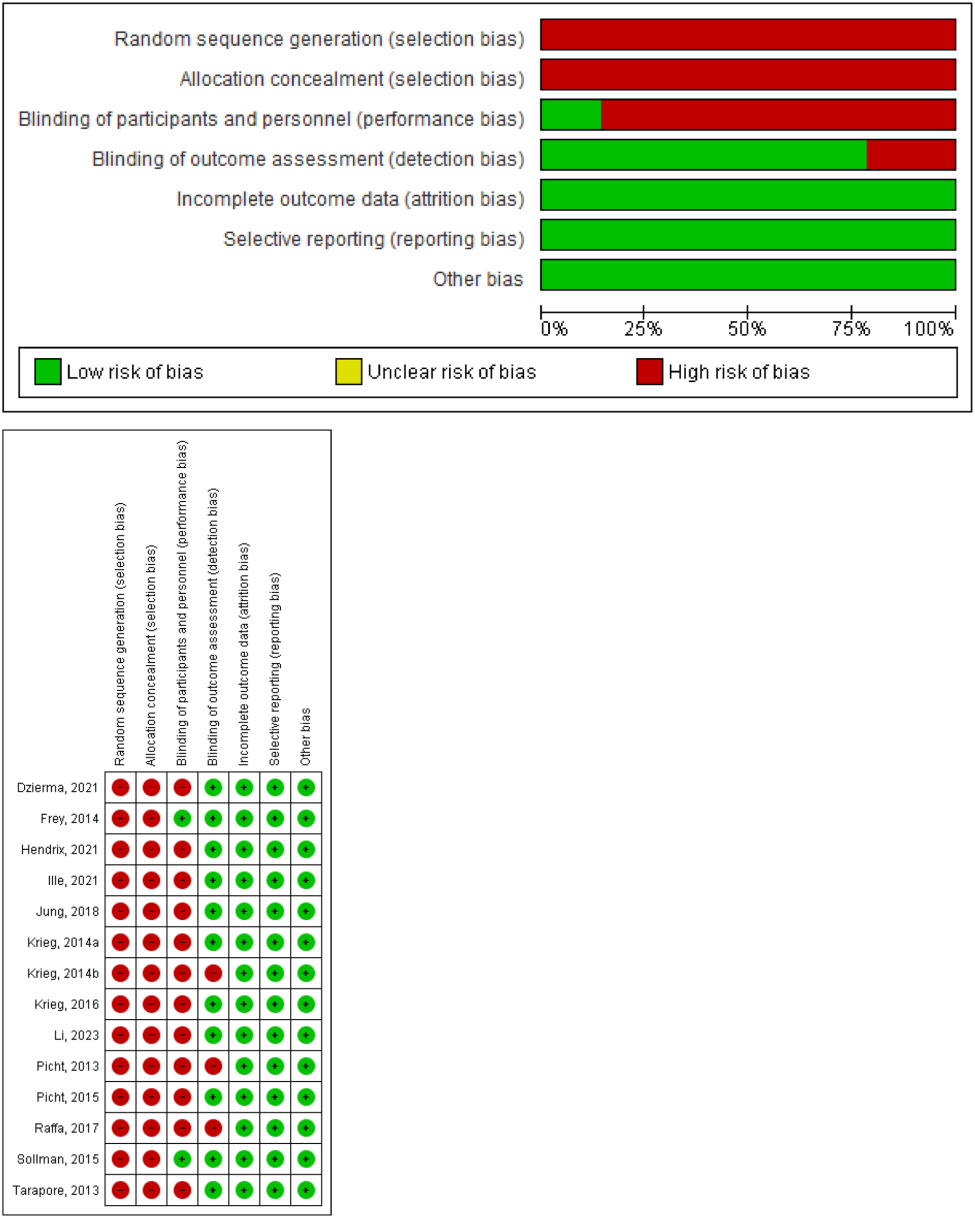
ROBINS-1 objective assessment

Fourteen studies were identified between 2010 and the 2023 interval. While this number is sufficient, most studies were not randomized and were not accompanied by either proper control or blinding. This may be understandable due to its invasive nature and the debilitating consequence should failure occur during the study; thus, objective assessments were never identified as exclusion criteria. Study assessment with the ROBINS-1 tool can be observed in Fig. 2.

### Studies characteristics

From all 14 studies, 1011 patients underwent TMS while 732 patients underwent DCS. This number were sufficiently and appropriately large. All these patients have record regarding pre-operative and post-operative clinical data. All of the study characteristics were presented in Table 2.

**Table 2:** Study charactheristics

Study	Country of origin	Patient pathology	Total patients		Result
			TMS	DCS	
Dzierma, 2021 [9]	Germany	Glioma, Brain metastases, Meningioma, Cavernoma	111	111	10 patient motor outcome deteriorated after 60 days in nTMS group and 9 patient in DCS group
Ille, 2021 [10]	Germany	Glioma	100	47	10 patients' lingual outcomes deteriorated after surgery in TMS and 4 patients in the DCS group
Jung, 2018 [11]	United Kingdom	Glioma, Metastasis, Epidermoid Cysts, Cavernoma	35	11	Two patients had permanent neurological deficits in the nTMS group compared with one patient in the motoric DCS group and one patient in the speech DCS group
Krieg, 2014 [12]	Germany	Glioma	20	12	8 patients had aphasia post-operation using TMS, while only 2 in the DCS group
Li, 2023 [13]	People Republic of China	Glioma	9	7	2 patients were classified as having aphasia in the TMS group, while 3 patients were classified as such in the DCS group
Picht, 2013 [14]	Germany	Glioma, Cavernoma	6	14	1 patient's aphasia was aggravated in the TMS group compared to 4 in the DCS group
Sollman, 2015 [15]	Germany	Glioma, Metastasis	25	25	2 patients' aphasia was aggravated in the TMS group compared to 4 in the DCS group
Tarapore, 2013 [16]	United States	Glioma	21	12	11 patients had speech arrest in the TMS group, compared to only 2 in the DCS group
Picht, 2015 [17]	Germany	Glioma	93	34	36 patients had either transient or permanent motoric deficits in TMS group compared to only 6 in control
Frey, 2014 [8]	Germany	Glioma, Metastasis	250	115	15 patients had either transient or permanent motoric deficits in TMS group compared to 10 in control
Hendrix, 2020 [18]	Germany	Glioma, Metastasis	105	105	15 patients had either transient or permanent motoric deficits in TMS group compared to 10 in control
Raffa, 2017 [19]	Italy	Glioma	16	9	15 patients had motoric deficits in TMS group compared to 10 in control however 3 patients become aphasia in TMS group compared to 1 in control
Krieg, 2016 [20]	Germany	Metastasis	120	130	7 patients had either transient or permanent motoric deficits in TMS group compared to 22 in control
Krieg, 2014 [21]	Germany	Glioma, Arteriovenous malformation, Cavernoma	100	100	29 patients had either transient or permanent motoric deficits in TMS group compared to 28 in control

### Motoric outcome

**Fig. 3:**
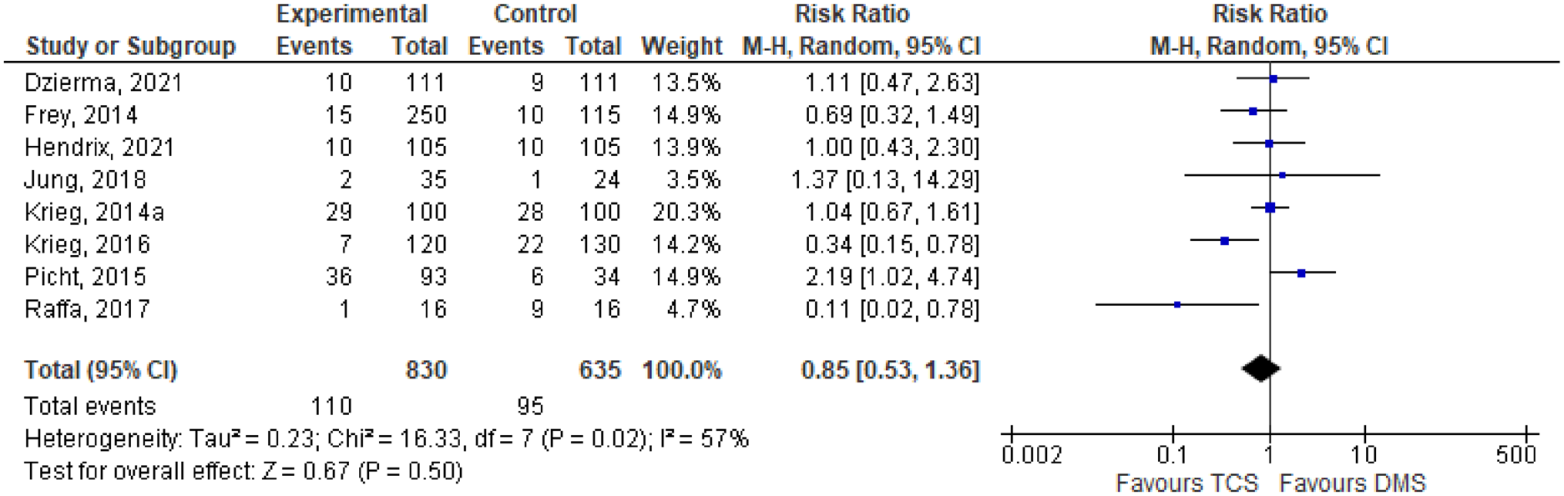
Forrest plot of motoric outcome comparison between DCS and TMS

Motoric outcome was assessed in 8 journals out of 14 available studies. The heterogeneity test through the I^2^ test revealed heterogenous data with I^2^ more than 50%, thus a random-effect model was used in the analysis. Considering that the variable assessed was motoric outcome, which was whether paresis happened after patient surgery, risk ratio was used as a parameter. Meta-analysis was then applied as a hypothesis test, which showed that TMS were not inferior compared to DCS in terms of motoric outcome, which was marked subjectively by diamond location and objectively through a p-value exactly 0.5. The Forrest plot will be presented in Fig. 3.

### Language ability

**Fig. 4:**
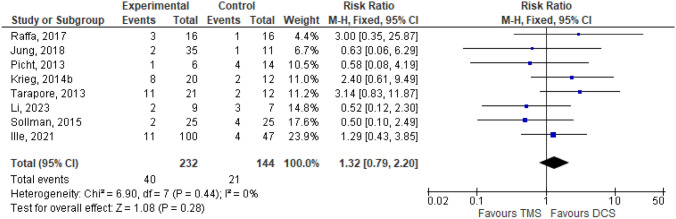
Forrest plot of lingual outcome comparison between DCS and TMS

Linguistic outcome was assessed in 8 journals out of 14 available studies. The heterogeneity test through the I^2^ test revealed homogenous data with I^2^ less than 50%, thus a fixed-effect model was used in the analysis. Considering that the variable assessed was the lingual outcome, which was whether aphasia happened after patient surgery, risk ratio was used as a parameter. Meta-analysis was then applied as a hypothesis test, which showed that TMS were not inferior compared to DCS in terms of aphasia occurrence after surgery, which was marked subjectively by diamond location and objectively through a p-value above 0.05. The Forrest plot will be presented in Fig. 4.

### Publication bias

**Fig. 5:**
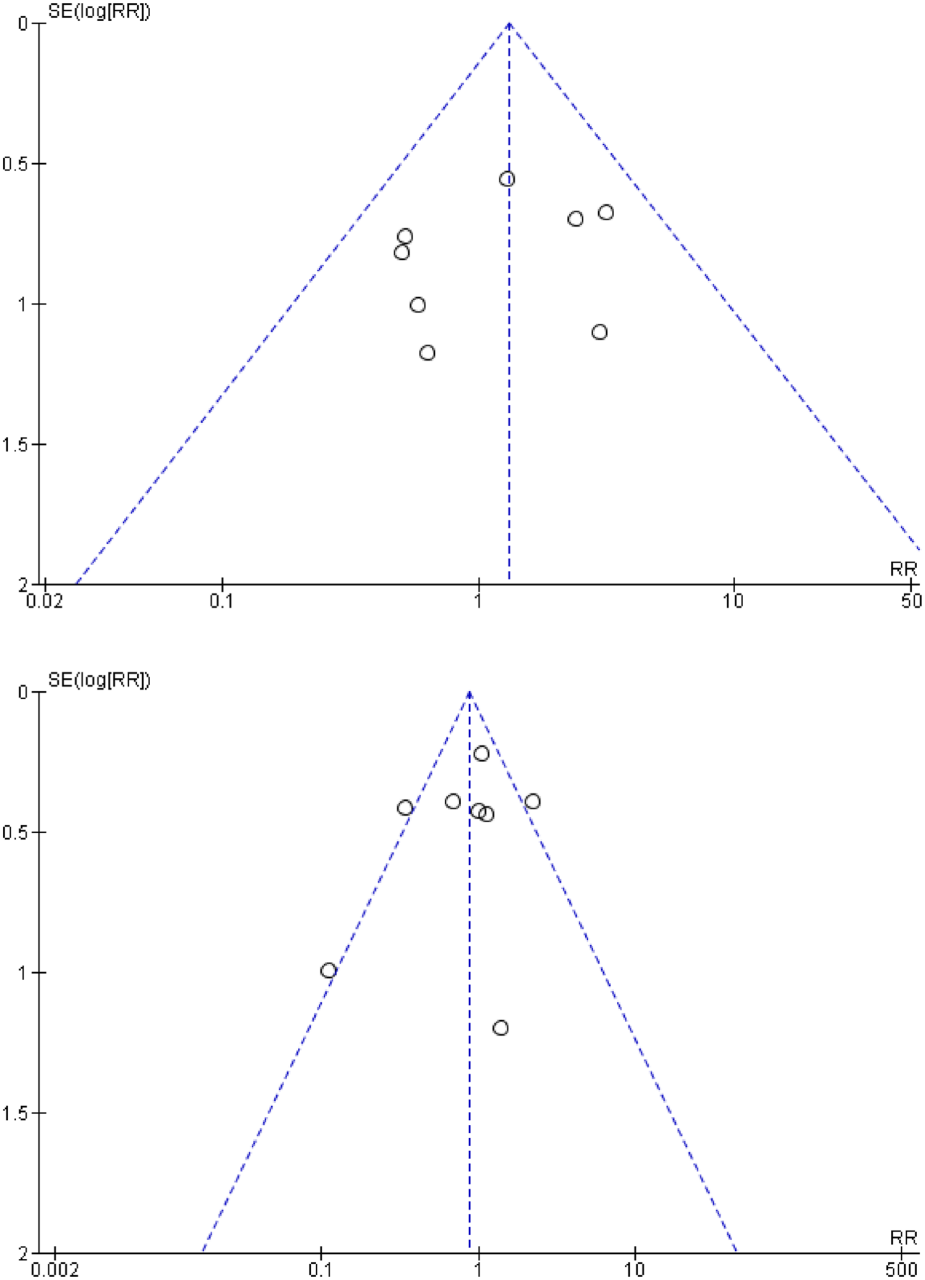
Funnel plot of studies; (above: language ability; below: motoric outcome)

To determine whether publication bias exist within the study, a subjective analysis with funnel plot were performed in which our graph shown 4 studies in the right treatment arm and 3 studies in the left treatment arm. This result was quite symmetrical and all studies were located inside the triangle thus we conclude that no significant publication bias exist in the study. In motoric outcome funnel plot analysis, while some studies were located outside of the triangle, no asymmetry were found. The funnel plots were presented in Fig. 5.

## Discussion

Currently in neurosurgery field of knowledge, resection of tumors in eloquent area has always been an interesting subject. Awake surgery was then developed to accommodate a safe surgery with low adverse outcome during resection of tumor. In the current era, the most precise way to localize the region is direct cortical stimulation (DCS) performed during awake craniotomy [22]. Intraoperative electrical stimulation of the motor cortex is a sensitive method for intraoperative mapping and monitoring of this region. Two different stimulation techniques have been established, the bipolar and monopolar techniques [7]. Such concept were not a new or foreign one. Sir Victor Horsley during its experimentation found that by electrically stimulating the cerebral cortex he could trigger extremity movement. Afterward this technique advances into a proper technique which neurosurgeon used to operate during operation of either epilepsy foci or brain tumor in eloquent area.

However, DCS were not without its own weakness. Due to its invasive nature, follow-up examination was not possible to assess plastic reshaping of cortical language function [22]. Awake surgery itself may not be available to all patient something due to factor as simple as patient refusal and inability to co-operate may hinder this technique as patient cooperation were vital to evaluate their language, memory and motoric skills. Several relative contraindications also exist such as obese patients, patients with a history of obstructive sleep apnea, difficult airways, and patients with chronic cough. Resection resulting in large blood loss is also not done under an awake craniotomy [23]. This may not be a problem in epilepsy surgery however this is particularly important in neuro-oncology as neuro-oncology procedure were associated with usually, longer duration of surgery thus more blood loss [24]. As all of these reasons were associated with its invasive nature, a less-invasive method would be necessary.

Navigated repetitive transcranial magnetic stimulation (nrTMS) has been increasingly used for preoperative language mapping in patients and motoric mapping. TMS was known as a less sophisticated and more stable physical method of mapping compared to MEG or fMRI while providing its own advantage over DCS. It is a unique method for detecting eloquent tissue directly comparable to intraoperative DCS and is a well-established tool, especially in neurology. TMS is used as a diagnostic and prognostic indicator for measuring central motor latency or detecting epileptic foci [25].

TMS employs electromagnetic induction principles. A magnetic field is created when an electric current is transmitted through a main coil, according to the concept of electromagnetic induction. When the magnetic flux goes to the secondary coil (neural tissue), a secondary electrical field is created, causing activation of the same. Neurons feature bent or curved axonal processes that run perpendicular to the magnetic field's lines of force. They function as secondary coils and hence experience electrical impacts. As a result, by altering the direction of current flow at HFs, quickly alternating magnetic fields can be created, stimulating the underlying neurons and their fibers. The phenomena of applying such stimulation in pulses is known as "pulsed EMF stimulation," and it generates persistent depolarization [26].

In neuro-oncology, navigated TMS (nTMS) is the main mapping tool. Navigated transcranial magnetic stimulation (nTMS) is a noninvasive imaging method for the evaluation of eloquent brain areas (e.g., controlling motor or language function). Transcranial magnetic pulses are delivered to the individual as a navigation system calculates the strength, location, and direction of the stimulating magnetic field. The locations of these pulses are registered on a magnetic resonance image of the individual’s brain. Surface electromyography (EMG) electrodes are attached to various limb muscles of the individual [27].

A systematic review and meta-analysis were of interest to us when compared to our review. Giovanni Raffa et al. performed a systematic review comparing TMS with control (whether the group underwent DCS or not) in 2019, which revealed that TMS were much better at providing a better motor outcome. These results were inconsistent with our review; however, we believe that both studies have merit. Our study focuses on eloquent area damage; thus, we include transient surgery-related paresis as these patients can be concluded to have side effects from eloquent area surgery. We also exclude groups that didn’t undergo DCS. In the end, we believe that these inconsistencies emphasize TMS's role as an adjuvant, not a replacement mapping technique [28].

Owing to all those facts, TMS may have its own advantage over DCS and may become a mapping alternative instead of DCS in conditions where DCS may not be used. However, it must be noted that our study was not without its limitations. The predominantly German-based study may indicate that this study might not be compatible with other populations. However, considering that the data collected from the UK, US, and China didn’t turn our cluster of data to heterogeneous data, it indicates that the physical properties remained the same. Second, the ROBINS-1 tool has revealed that most studies are generally weak in evidence power and bias risk. However, as emphasized previously, neuro-oncology is a particularly difficult area in research due to its ethical implications, and these studies might as well be the best studies there are considering their ethical problems.

## Conclusion

TMS is a noninvasive imaging method for the evaluation of eloquent brain areas that is not inferior compared to the invasive gold-standard imaging method (DCS). TMS could be considered an evaluation tool in neuro-oncology patients, an alternative mapping method in which awake surgery was contraindicated, and an adjuvant to DCS to further improve surgeon visualization. It needs to be emphasized that TMS should not become a replacement mapping method and should be used as an adjuvant together with the gold standard DCS. TMS's role as a replacement is appropriate only when awake surgery isn’t available.

## Data Availability

As this article is a systematic review, no datasets were necessary and available.
